# Conscience

**Published:** 1889-10-26

**Authors:** 


### The Hospital

A Weekly Institutional Journal of

Science, Medicine, Nursing, and Philanthropy.

*For Hospitals, Asylums, Medical Practitioners, Students, Nurses, Families, Parishes, Congregations, and the Charitable Public.*

Vol. VII. No. 161.] [October 26, 1889.

### Conscience.

The careful observer of English character and life becomes gradually but firmly convinced that the bulk of his countrymen possess in a very marked degree that highest product of mental development, a conscience. It is not always an enlightened nor always a sensitive conscience; but a true and authoritative conscience it certainly is, when it can be got at. If this were an academic essay we might here cite historical and other proofs of our thesis. But that would be carrying us away from our immediate purpose, which is rather to "get at" the conscience of everyone of our readers than to convince him that he possesses such a faculty. "If a man would speak faithfully, he must needs believe firmly," wrote Carlyle. There is no merit in holding a deep and earnest conviction as to the use and value of the hospitals of this country if a man have had prolonged experience of them. It is impossible for any fairminded person to be much in and about any of the British hospitals without coming to the conclusion that they are institutions where practically nothing but the best work is done in the best way. The work is of such a kind that every person of ordinarily good disposition who comes much into contact with it cannot help feeling an earnest desire to take part. Those who do take part, whether as medical men, nurses, subscribers, or managers, almost invariably find themselves improved in character by it, and they experience a deep and permanent satisfaction in it

Hospital supporters must be regarded as converts and adherents to a faith or "cult," the cult of "helping the helpless and comforting and healing the sick." The mere sound of the words moves the heart. Those who not only admire the motto of the cult, but give themselves up enthusiastically to the practice of its faith, are like other religious enthusiasts, they feel an intense desire to make converts and an equal eagerness to raise the tone and character of their associates on the peculiar lines and principles of the cult. Now, whether the cult of "helping the helpless and comforting and healing the sick" be regarded as a religion in itself, or as a part of Christianity, or of the "Religion of Humanity," in either case it is a rational, a useful, and a noble faith. It offers no marks for the arrows of the sceptic, no insoluble mysteries for the metaphysician to founder his bark upon, no ecclesiastical offences for the schismatic to rail at. But that is saying a great deal for it; it is saying that the faith is worthy of all acceptation; that, in fact, he who does not accept it, at least in approval and sympathy, is an ill-conditioned person.

Has every reader gone with us so far? Then we turn round upon him with the practical question, is he himself at the present moment a subscriber to, or in any way a helper of, the hospitals? If he is, what he needs to do is to ask himself whether he, as a convert, puts all the heart, soul, and practical endeavour into his hospital religion that the case demands; and if he does not, let him honestly admit the fact, and, like a man of sense and worth, begin forthwith to satisfy his conscience by better doing his duty. But many people who read this may not belong to the "hospital faith" at all, and may never have given a penny to, or in any other way helped a single hospital. In spite of that, it is possible that such people have done their duty in the world according to their lights by helping other good objects. But what we wish to point out, and what ought to be pointed out, is the relative position of hospitals with regard to other good and charitable institutions. Where do they stand as compared with similar charities? Are they at the foot of the hill of merit, or are they halfway up, or are they at the very summit, so that in regard to their acknowledged value they can hardly ascend any higher? Undoubtedly, they are at the apex of the pyramid of benevolent institutions. In saying this, we are not depreciating orphanages, asylums, convalescent homes, or any of the manifold forms in which British benevolence displays itself. The object is to show that hospitals have a unique place among charitable works, and that therefore they demand a unique consideration.

The man of intelligence recognises that it is his duty to make the best use he can of his money. This holds good as truly of money devoted to charity as of all money. It is as possible to misuse resources on so-called charitable objects as on gaming, drinking, or any other vice. It may be said with truth that if a man will exercise the smallest amount of discrimination, it is not possible for him to waste money in giving to a hospital. That is to say, the meritorious hospitals are so numerous as compared with the few that are not meritorious, that any man of good sense can find out by the most casual enquiry a score of hospitals which thoroughly deserve his utmost support. There is therefore no excuse for the carelessness of the careless man who gives foolishly, still less for the selfishness of the selfish who does not give at all. An honest conscience will not approve of the neglect of hospitals on either of these very common grounds. The question has to be looked squarely in the face. We make no apology whatever for trying to compel attention to it. It is an undoubted fact that there are thousands, probably millions, of prosperous people who never from January to Decem-

ARMY MEDICAL LIBRARY

ber disburse one single penny in charity. Such people are guilty of a wicked neglect of duty, and their neglect not only injures the sick poor who use the hospitals, but injures themselves in a tenfold greater degree. Whoever, by persuasion or reproof, or any other justifiable means, succeeds in forcing home upon the consciences of the indifferent the irresistible claims of charity renders a great service to hospitals, and a still more conspicuous service to the careless, who are thereby awakened to a reasonable sense of duty.

The cynical and the cold-hearted may be full of reasons why they should not help hospitals, at any rate at the present time. They always have plenty of reasons for doing everything they like, and leaving undone everything they don't like. What they don't like is denying themselves a single indulgence for the good of others. They will explain most cunningly that the poor might have been quite as well off as they themselves are if they had only used their resources with the same discretion. That is adding hypocrisy to meanness, for they do not believe in their own explanations. No doubt many of the poor are the worst possible managers of money; but the better among them often make one shilling do the duty of ten. The shifts and cunning expedients which many a cottage mother employs would make the reputation of a Chancellor of the Exchequer a thousand times over. In Yorkshire, a short time ago, a farm labourer was summoned for non-payment of his doctor's bill. He was a man with a family. The judge of the county court asked what his wages were. "Two shillings a week," answered the man; two shillings a week and his food. The judge did not believe him, and enquired who his master was. "There he is, in court," said the labourer; and the master could not deny that the facts were exactly as stated. Will any man or woman assert that that man was able to provide against sickness, or to pay a doctor's bill for the sick members of his family? And yet were not the members of his family sure to be sick; sick for want of food, and clothing, and shelter, if for no other reason? What can a man do for his family with two shillings a week or even with six times two? If he feeds and clothes and sends them to school and puts a roof over their heads with that sum, he or his wife works a miracle compared with which some of the wonders of science are but as child's play.

Some people expect from poor, ignorant, and half-fed labourers a standard of practical sense and moral excellence which they never dream of looking for in the wealthy and educated classes. Such people are either uncommonly stupid or exceedingly disingenuous. Let them bring common-sense and conscience to the consideration of the question of the poor and the hospitals, then they will soon arrive at juster and more practical conclusions. What the present supporters of charitable institutions wish to see in outsiders is an honest desire to examine the whole question fully and fairly. Such an examination can only have one result. Taken as a whole, the hospital system of this country is more than admirable. If a man have had no conscience before, a thorough consideration of the needs and value of hospitals will create a conscience in him. It is the steady support of instructed consciences that hospitals wish for, not the mere spasmodic help of fitful sentimentality. The regular income maintains the family, not the large wages of the harvest and the hop-picking season. So the daily flow of moderate subscriptions into the hospital treasury, from those who recognise the claims of duty is recognised as the backbone and the life-blood of the institution. A quarter of an hour every week given by each individual to the careful study of the hospital question would convert medical charities into perfect models of economy and efficiency. The magnitude of the subject may fairly claim that measure of consideration from every adult man and woman.

